# Functional Recovery of a Locomotor Network after Injury: Plasticity beyond the Central Nervous System

**DOI:** 10.1523/ENEURO.0195-18.2018

**Published:** 2018-07-11

**Authors:** Joshua G. Puhl, Anthony W. Bigelow, Mara C. P. Rue, Karen A. Mesce

**Affiliations:** Graduate Program in Neuroscience, Departments of Entomology and Neuroscience, University of Minnesota, St. Paul, MN

**Keywords:** CPG, dopamine, homeostatic plasticity, locomotion, spinal cord injury, crawling

## Abstract

Many animals depend on descending information from the brain for the initiation and proper execution of locomotion. Interestingly, after injury and the loss of such inputs, locomotor function can sometimes be regained without the regrowth of central connections. In the medicinal leech, *Hirudo verbana*, we have shown that crawling reemerges after removal of descending inputs. Here, we studied the mechanisms underlying this return of locomotion by asking if central pattern generators (CPGs) in crawl-recovered leeches are sufficient to produce crawl-specific intersegmental coordination. From recovered animals, we treated isolated chains of ganglia with dopamine to activate the crawl CPGs (one crawl CPG per ganglion) and observed fictive crawl-like bursting in the dorsal-longitudinal-excitor motoneuron (DE-3), an established crawl-monitor neuron. However, these preparations did not exhibit crawl-specific coordination across the CPGs. Although the crawl CPGs always generated bidirectional activation of adjacent CPGs, we never observed crawl-appropriate intersegmental phase delays. Because central circuits alone were unable to organize crawl-specific coordination, we tested the coordinating role of the peripheral nervous system. In transected leeches normally destined for recovery, we removed afferent information to the anterior-most (lead) ganglion located below the nerve-cord transection site. In these dually treated animals, overt crawling was greatly delayed or prevented. After filling the peripheral nerves with Neurobiotin tracer distal to the nerve-root lesion, we found a perfect correlation between regrowth of peripheral neuronal fibers and crawl recovery. Our study establishes that during recovery after injury, crawl-specific intersegmental coordination switches to a new dependence on afferent information.

## Significance Statement

In the field of motor control, a pressing issue to resolve is how the central and peripheral nervous systems become retuned and work together to establish functional locomotor patterns after descending signals are chronically removed after injury. The work we have presented, in the medicinal leech, clarifies that central pattern generator (CPG) neural networks are highly plastic in their ability to incorporate different timing elements for their intersegmental coordination after descending-coordinating information is removed. Essentially, the crawl CPGs switch their dependence on descending-timing cues to those originating from proprioceptive neurons in the muscular body wall. Results from our experimentally tractable preparation may provide a platform to guide future studies of locomotor recovery in vertebrate animals after spinal cord injury.

## Introduction

After significant injury to the spinal cord of vertebrates or the ventral nerve cord of invertebrates, motor activity distal to the site of injury is impaired or lost (Rossignol et al., 1996; Puhl and Mesce, 2010; Rasmussen and Sagasti, 2017). Under certain circumstances, some recovery of reflexive behaviors and locomotion can be achieved. Several studies have reported that motor recovery is realized via the regrowth and reconnection of neural fibers across the site of injury (Parker, 2017; Rasmussen and Sagasti, 2017; Joven and Simon, 2018). In contrast, and somewhat more enigmatic, are findings that the restoration of motor function can occur without the successful reconnection of damaged central pathways (Edgerton et al., 2004; Sakurai and Katz, 2009; Rossignol and Frigon, 2011; Harley et al., 2015). Thus, a critical problem in the neurosciences is to understand how a neural system can functionally recover when its higher-order descending inputs, critical for action selection and movement, are permanently lost.

It is clear that regions of the central nervous system (CNS) that are orphaned from higher-order areas must undergo some sort of significant physiologic and/or anatomic plasticity to regain their former functional state. Changes of this nature have been deemed compensatory or “homeostatic plasticity” (Turrigiano, 1999, 2012; Marder, 2011). In some cases, this plasticity is detrimental to overall functional recovery (e.g., Beauparlant et al., 2013); however, it often leads to the reestablishment of the operation of a given cell or circuit. Although the phenomenon of homeostatic plasticity underlying functional recovery after injury has been studied in a number of organisms across taxa (Muller et al., 1987; Edgerton et al., 2004; Ivanenko et al., 2013; Parker, 2017), fewer studies exist that describe the cellular underpinnings of how motor neural networks might achieve their new operational states (e.g., McClellan et al., 2008; Pozo and Goda, 2010; Husch et al., 2012).

Our work investigates the cellular mechanisms of locomotor recovery in the European medicinal leech, *Hirudo verbana*. For many decades, the medicinal leech has served as a well-established model system for revealing the network and cellular bases of behavior, including swimming and crawling (Kristan et al., 2005). We have shown previously that leeches can recover their ability to crawl after a complete transection of the ventral nerve cord below the brain, which serves to remove identified descending fibers normally necessary for crawl initiation and coordination (Harley et al., 2015). Crawling is the terrestrial form of locomotion and is defined, at its core, as alternating elongations and contractions of the body with associated attachments of the anterior and posterior muscular suckers (Stern-Tomlinson et al., 1986). During each whole-body elongation or contraction, individual segments of the body (all 21) become active such that a highly coordinated wave, directed caudally, is propagated along the entire body, allowing the animal to propel itself forward along its substrate. Crawling is known to be centrally generated (Eisenhart et al., 2000), with each segmental ganglion (one per segmental division of the body) containing a complete central pattern generator (CPG) for crawling (Puhl and Mesce, 2008). Maintenance of the intersegmental coordination among the 21 segments is orchestrated by a cephalic command neuron named R3b-1 and is biased by DA (Puhl and Mesce, 2008, 2010; Puhl et al., 2012).

After complete transection (i.e., injury) of the CNS, leeches immediately lose their ability to generate crawl-specific movements (Harley et al., 2015), presumably because R3b-1 signaling is interrupted. Several days later, however, rudimentary crawl-like movements begin to be expressed, although proper intersegmental coordination is lacking. After several weeks, coordinated crawling movements are reestablished and overt crawling is difficult to distinguish from uninjured animals. This crawl recovery is not reliant on the regrowth and reconnectivity of the damaged CNS fibers; in fact, additional experimental efforts have been made to prevent reconnections from forming (Harley et al., 2015). Thus, some type of homeostatic plasticity must be in play to enable the 21 independent crawl CPGs to regain their coordination (i.e., normal phase relationships) with each other. Furthermore, Harley et al. (2015) reported that the ganglion (crawl CPG) immediately posterior to the site of transection, termed the ‘lead’ ganglion, became critical for recovered crawling. For example, if the lead ganglion were removed after crawl recovery, a second round of recovery was needed, with a similar but somewhat longer time course. Given the remarkable ability of the leech CNS to undergo wide-scale homeostatic plasticity, our study set out to determine the neural mechanisms by which the crawl CPGs were able to regain their intersegmental coordination in the absence of descending inputs.

## Materials and Methods

### Animals and solutions

Adult (hermaphroditic) medicinal leeches (*Hirudo verbana*) were initially housed in a glass aquarium filled with locally sourced natural spring water (i.e., pond water equivalent) at room temperature (22–24°C). Leeches were obtained from Niagara Medical Leeches and ranged in mass from 2 to 3.5 g.

Before surgery or dissection, leeches were anesthetized by immersion in ice-cold leech saline or crushed ice for 5 min. Normal leech saline contained (in mm): 116.0 NaCl, 4.0 KCl, 1.8 CaCl_2_, 1.5 MgCl_2_, 10.0 dextrose, and 10.0 Trisma preset crystals, pH 7.4 (all components from Sigma Aldrich), dissolved in deionized water (adapted from Nicholls and Baylor, 1968). To induce ganglia to generate the fictive crawling *in vitro* 50-100 µM dopamine (DA), HCl (Sigma Aldrich) was added to normal leech saline and used within 1 h (Puhl and Mesce, 2008). Isotonic Millonig’s buffer contained (in mm): 13 NaH_2_PO_4_, 86 Na_2_HPO_4_, and 75 NaCl. Hypotonic Millonig’s buffer did not contain NaCl.

To induce fictive crawling *in vitro*, we bath applied DA to entire preparations or to a subset of ganglia (Puhl and Mesce, 2008). The DA was applied either by superfusion or by replacing the bath solution manually with 3–5 50% bath volume changes. Superfusion was established by gravity-fed inflow and passive suction outflow at a rate of ∼1–3 ml/min.

### Surgeries and dissections

The ventral nerve cord of *H. verbana* contains an anterior compound cephalic ganglion (i.e., the brain), 21 midbody ganglia (one per segment, designated M1–M21), and a compound terminal ganglion. These structures are joined by bundles of neuronal fibers called connectives, which contain two lateral hemi-connectives and a median Faivre’s nerve. Two general types of surgical manipulations were performed. The first type entailed the complete transection (cutting) of the nerve cord, which involved completely severing all components of the connective between two ganglia. For crawl-related surgeries, the transection was made between ganglion 2 (M2) and ganglion 3 (M3). The second type was the bilateral denervation of the nerve roots, which involved severing the anterior and posterior nerve roots on both the left and right sides of an individual ganglion. We performed surgical procedures involving nerve root denervations only, connective transections only, and a combined procedure including both a connective transection and nerve root denervation. The surgical procedures involved anesthetizing the leech on ice for 5–10 min, pinning it in a wax-bottom dissection dish ventral side up, and making a small 2–4-mm midline, ventral incision through the cuticle and ventral muscle layers, exposing the CNS. Thin 200-µm pins were placed through the lateral edge of the body to help keep the incision site open while the appropriate CNS structures were severed using a fine scissors, taking care to minimize damage to the deeper structures of the body. After surgery, the incisions on some animals were sutured closed using Ethilon size 6-0 polyamide thread (Ethilon). All leeches were housed individually in a plastic chamber (∼15 × 15 cm; Glad brand) filled with pond water.

**Figure 1.**
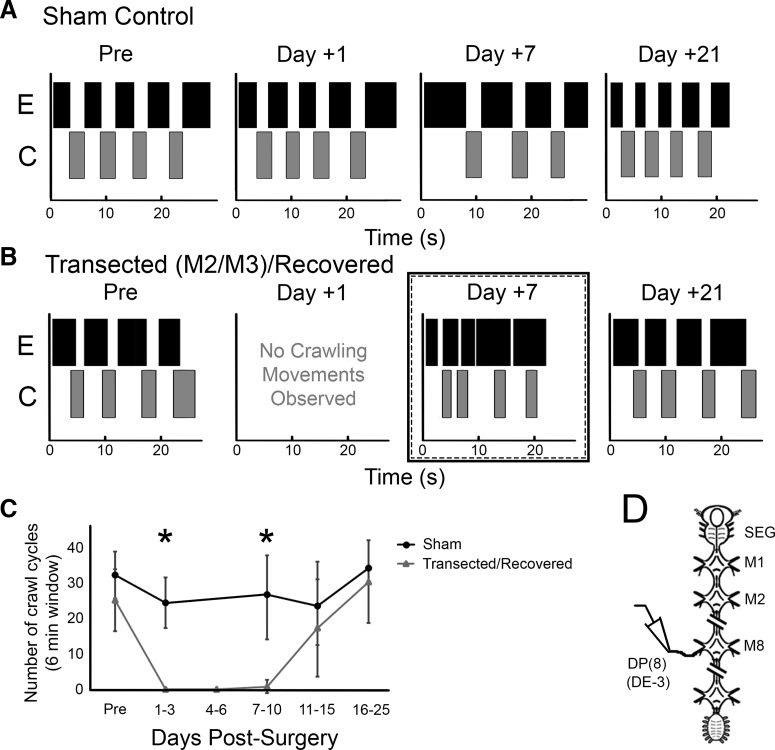
Overt crawling behavior after complete transection of the M2/M3 interganglionic connectives. ***A***,***B***, Movement raster plots depicting elongation (E, black boxes) and contraction (C, gray boxes) movements over time for individual leeches that received a sham surgery (***A***) and an M2/M3 transection surgery (***B***). Movements are shown for time points before the surgery (Pre), 1, 7, and 21 days after surgery. ***C***, Graph of crawling activity at several time points after surgery for a random subset of the leeches used in Figs. 2–6 (*n* = 10 for each group). Error bars denote the standard deviation. *, statistically significant differences (*p* < 0.01). ***D***, Schematic depicting a truncated view of the CNS of a leech. All nerve recordings showing fictive locomotor activity were obtained from the dorsal posterior nerve root (DP nerve), which contains the axon of the motoneuron DE-3. Although all 21 segmental ganglia have DPs, only the DP associated with mid-body ganglion M8 (DP(8)) is illustrated here. Diagonal hash marks denote ganglia that were removed to simplify the cartoon.

In preparation for electrophysiological recordings, CNS tissues were dissected from the leech body along with both the left and right dorsal posterior nerves (DP nerves) on 3–4 ganglia. The connectives were left intact and undamaged in between the dissected ganglia. Tissues were pinned down in a Sylgard-lined (Dow-Corning) recording dish initially filled with normal leech saline. The term “whole nerve cord” preparation describes the dissection of the entire CNS and comprised the cephalic ganglion, all 21 midbody ganglia, and the terminal ganglion. The term “whole recovered cord” refers to dissection of all the CNS structures posterior to the site of complete nerve cord transection (usually from M3 to the terminal ganglion) in a leech that demonstrated full recovery of crawling behavior after injury (see below and Fig. 1; Harley et al., 2015). A 5-ganglion “chain” preparation consisted of 5 ganglia from the anterior region of the CNS (M3–M7) or the middle-body region (M8–M15). In some experiments, the saline solution surrounding one or several ganglia was isolated by a petroleum jelly well to facilitate focal application of DA.

### Behavioral monitoring and peripheral nerve backfills

Before surgery and every 2–3 days after surgery (for up to 45 days), leeches were placed in a 20.3-cm-diameter circular plastic chamber with low water levels, and video was recorded for 6 min similar to the methods of Harley et al. (2015). Crawling behavior is defined as mentioned above (Stern-Tomlinson et al., 1986). Furthermore, movements of the body segments had to be coordinated in an anterior-to-posterior direction. In CNS tissues dissected from the body, the neural correlates of locomotion were monitored via extracellular recordings of 2–4 DP nerves according to the methods of Puhl and Mesce (2008). As previously described, to be deemed fictive crawling, DE-3 crawl burst periods (in the DP nerve) had to be between 5 and 30 s. Intersegmental delays (burst onset to burst onset) of <2 s/adjacent segment were used. These intersegmental delay values are more permissive than our previous work (Puhl and Mesce, 2008, 2010) to account for any potential delay or variability added due to the injury or recovery process.

In a subset of preparations, nerve roots were backfilled with Neurobiotin tracer (Mesce et al., 1993). Briefly, a leech was pinned ventral side up in a dissection chamber and anesthetized on ice for 5–10 min. A ventral, midline incision was made using a sharp blade through the cuticle and muscle layers. Using fine scissors, a patch of cuticle was separated from the muscle layers surrounding the nerve to be filled and pinned down. Muscular and connective tissues were carefully separated from a given peripheral nerve. The ending of the nerve was placed inside a petroleum jelly well filled with a solution of ∼5% Neurobiotin (w/v) in 50% leech saline/50% deionized water. The preparation was surrounded by leech saline and covered. It was incubated at room temperature for 0.5–2 hours and then placed at 4°C for 40–60 hours. After incubation, the ganglion attached to the nerve and surrounding CNS structures were dissected, fixed in 4% paraformaldehyde in Millonig’s buffer at room temperature for 1 h, rinsed several times in fresh Millonig’s buffer, and transferred into Cy3-conjugated Streptavidin in 0.1% Triton X-100 for 24–72 hours at 4°C. It was then rinsed several times in hypotonic Millonig’s buffer, dehydrated, cleared with methyl salicylate, and mounted between two coverslips in DePeX mounting medium (Electron Microscopy Sciences). The Cy3 fluorophore was visualized and imaged using a Nikon N1 laser-scanning confocal microscope, and the images were prepared using ImageJ and Adobe Photoshop.

### Electrophysiological recordings

We recorded extracellular unit activity from DP nerves of dissected CNS tissues, *in vitro*, using custom-made plastic suction electrodes (∼50-µm tip diameter) attached to an A-M Systems model 1700 AC-coupled differential amplifier. The signals were digitized at 10 kHz using a Digidata 1440A digital-to-analog converter (Molecular Devices) and stored on a computer. Within the DP multi-unit recordings, we focused our analyses on the largest unit in DP recordings (Fig. 1*D*), which arises from the dorsal longitudinal excitor 3 motoneuron (DE-3; Kristan et al., 1974). DE-3 is rhythmically active during the contraction phase of crawling, and thus served as a convenient monitor of fictive crawling in isolated CNS preparations (Eisenhart et al., 2000; Puhl and Mesce, 2008).

### Data analysis and statistics

The periods and intersegmental phase delays of DE-3 bursting were determined either by using custom scripts written in Matlab (Mathworks) or manually by identifying the beginning and ending of DE-3 bursts using ClampFit analysis software (Molecular Devices) and calculating the parameters in Microsoft Excel or Matlab. Mean periods and the observational frequency of coordinated events were reported as mean ± standard error of the mean. In Fig. 1, the number of crawl cycles was reported as mean ± standard deviation. Nonparametric, two-tailed statistical comparisons (Mann–Whitney–Wilcoxon and Kruskal–Wallis H test methods) were conducted in either R (R core team, 2017) or Python using a confidence level of α = 0.05.

## Results

We tested the hypothesis that the recovery of crawling arises exclusively from plastic events originating within the CNS such that the crawl CPGs would become competent to produce crawl-specific interoscillator coordination. Furthermore, we reasoned that afferent information would not be a critical factor in the recovery process, and thus predicted that *in vitro* fictive crawling would be obtainable from recovered animals as it is in control animals. Our study thus required the generation of newly transected and recovered animal subjects for subsequent analysis of fictive crawling and other locomotor activity *in vitro*.

For better ease of nerve cord manipulation and assessment of any potential nerve fiber reconnectivity, leeches were transected between M2 and M3. Because Harley et al. (2015) performed nerve cord transections primarily between the SEG and M1, we tracked and analyzed the recovery process of the M2/M3 transected leeches in comparison to our previous study. Collectively, full recovery took place 19 ± 4.2 days (*n* = 20) after the transection surgery, a time course similar to Harley et al. (2015). We observed the body movements of these leeches, along with leeches receiving a sham surgery, in low water conditions. Fig. 1 describes the crawling movements of a sham (Fig. 1*A*) and a transected/recovered (Fig. 1*B*) leech. Before the surgery (pre), both leeches produced alternating elongation and contraction movements of the body with crawl-appropriate coordination among the segments. One day after the transection surgery (day +1), the sham control leech continued to show normal crawling behavior, while the transected one exhibited no crawling. After 1 week (day +7), the transected leech began to show elongation and contraction body movements, but they were not alternating, nor did they propagate from the anterior to posterior region of the body (data not shown). By 21 days post-surgery, the transected leeches exhibited normal-looking crawling. We counted the number of crawl cycles of a subsample of the transected and sham leeches (Fig 1*C*; *n* = 10 leeches for each). Not surprisingly, in the sham-control leeches, the ability to crawl was unaffected by the surgical procedure. In the transected group, none of the leeches exhibited any crawling within 3 days of surgery, and only one was crawling by day 6. By day 15, >70% were crawling, and all expressed coordinated crawling by days +16 to 25. The number of crawl cycles between the sham and transected/recovered leeches was significantly different for the post-surgery days 1–3 and 7–10 time points (*p* < 0.01 for both).

**Figure 2.**
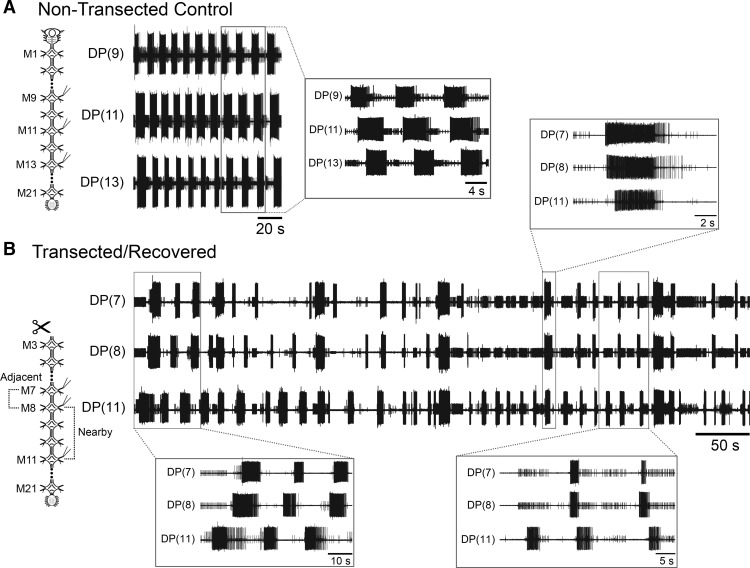
Fictive crawling-like activity of an *in vitro* whole recovered nerve cord treated with dopamine. ***A***, Left, schematic of a nontransected control isolated nerve cord preparation. Traces, three dorsal posterior (DP) nerve root recordings from a control nerve cord treated with 100 µM dopamine. Inset, time-expanded view of several bursts from the traces showing intersegmental coordination among the ganglia. Boxes denote the source of the time-expanded traces. ***B***, Left, schematic depicting a recovered whole nerve cord (M3-TG) from an M2/M3 transected leech that recovered crawling behavior. Traces, three DP nerve recordings of a recovered whole cord treated with 50 µM dopamine. Insets, time-expanded views of several time-spans of the recording presented. Boxes denote the sources of the time-expanded traces. Note: The largest unit in all DP recordings is the dorsal longitudinal excitor-3 motoneuron (DE-3).

We then investigated whether nerve cords dissected from the body, thus lacking functional afferent inputs, were capable of generating the neural instructions necessary to generate coordinated fictive crawling. We monitored extracellular neural activity in the DP nerves of several ganglia distributed along dissected nerve cords (Fig. 1*D*) treated with the biogenic amine DA. In uninjured cords and acting in concert with R3b-1, DA is sufficient to activate every segmental crawl CPG and induce appropriate oscillator phase delays (Puhl and Mesce, 2008). Fig. 2*A* is a representative recording of three DP nerves from an uninjured control leech treated with 100 µM DA. The motoneuron DE-3, the largest unit in a DP recording, generated bursts of action potentials in each of the DP recordings. Motoneuron DE-3 is active during the contraction phase of fictive and overt crawling. The onset of DP bursts was delayed between successive posterior segments, thus establishing an anterior to posterior metachronal wave of excitation (Fig. 2*A*, blow-up), an essential element of established fictive crawling in control CNS preparations (Eisenhart et al., 2000). Fig. 2*B* shows a typical result when 50 µM DA was applied to a whole nerve cord preparation from a leech that expressed overt crawling (i.e., complete nerve cord posterior to the site of transection). In these preparations, we recorded from three DP nerves. One pair of recordings was from ganglia adjacent to each other (M7 and M8 in Fig. 2*B*), and a third nerve from a ganglion located several segments away, completing a second pair of “nearby” recordings (M8 and M11). We observed rhythmic DE-3 bursting in all of the DP nerves, indicating that the crawl CPGs were functional without sensory inputs in the injured but recovered condition. What was absent from these recordings, however, was any sustained crawl-specific intersegmental coordination. In the bottom left inset, DE-3 bursting was observed in all three channels at the same time, but the intersegmental delays appropriate for fictive crawling were not present. In the bottom right inset, bursting was again observed, but there was not a 1:1 correlation of bursts in all channels recorded, nor was the onset of DE-3 bursting propagating in a caudal crawl-like manner. The top inset of Fig. 2*B* shows a singular burst, which did exhibit coordination that was appropriate for crawling. These coordinated bursts, however, were not sustained in subsequent bursts and thus were deemed an isolated coordinated event.

**Figure 3.**
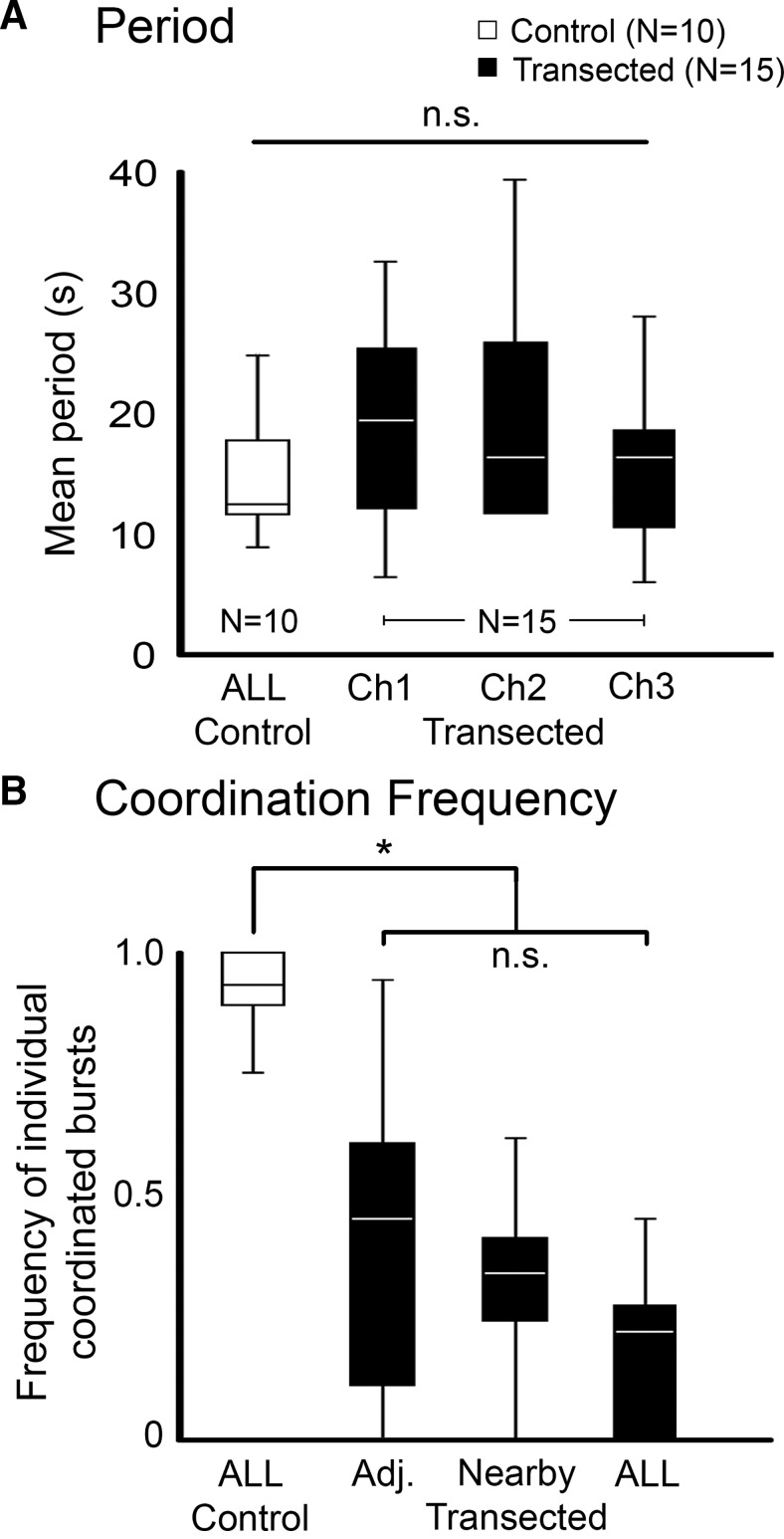
Quantification of whole recovered cord data. ***A***, Boxplots of the mean cycle period of DE-3 bursting of isolated whole nerve cords treated with dopamine. White boxes are from controls (*n* = 10) and black boxes from M2/M3 transected and recovered leeches (*n* = 15). Note: The periods of the three DP nerves in the controls were isochronous, so only one box is shown. For preparations from transected and recovered leeches, each channel analyzed is shown. Lines within the boxes denote the median, and the box range denotes the 25th–75th percentiles. Error bars denote the entire range of the dataset. No statistical differences were found between the groups (n.s., Kruskal–Wallis *H* test, *p* > 0.5) ***B***, Boxplots showing the frequency of observing crawl-specific coordination of individual DE-3 bursts. White boxes are from untransected control preparations. The black boxes depict the frequency of coordination between two adjacent ganglia (Adj.), ganglia within 2–3 segments from one another (Nearby), and among all ganglia recorded in a preparation (ALL). Box plot parameters are the same as in **A**. *, statistically significant difference between the control and transected/recovered groups (*p* < 0.01). No significant differences were observed among the transected recordings (n.s.; Kruskal–Wallis *H* test, *p* > 0.5).

We analyzed the DA-induced DE-3 (DP) bursting in multiple control (*n* = 10) and recovered (*n* = 15) nerve cords. Fig. 3*A* reports the cycle periods for these experiments. The mean period duration for control preparations was 14.3 ± 5.2 s. The mean period observed in the DP nerves of recovered whole cords was 17.9 ± 8.1 s. The mean periods of both the controls and the recovered preparations were within the normal range for DA-induced fictive crawling (Puhl and Mesce, 2008) and were not significantly different from one another (*p* > 0.5). Importantly, the range of periods across the different DP recordings in the recovered whole cord preparations was greater than in the control nerve cords. In control cords, the periods among the three recording channels were isochronous. In the recovered whole cords, there was an increased variability in periodic bursting across the different segments, which appeared to contribute to the lack of coordination. This lack of coordination was more obvious when we analyzed the frequency of observing crawl-specific coordination between recording pairs (Fig. 3*B*). In control nerve cords, the frequency of intersegmental coordination was 0.92 ± 0.09; the bursting was almost always coordinated. In recovered whole cords, the coordination frequency was 0.40 ± 0.31 for adjacent ganglia and 0.32 ± 0.16 in nearby ones. The observational frequency of coordination in the controls compared to the recovered preparations was significantly different (*p* < 0.01). Within the recovered whole cords, the frequency was slightly higher in adjacent ganglia compared to those farther away, but the difference was not significant (*p* > 0.5). The frequency of observing crawl-specific intersegmental coordination among all three recordings was 0.18 ± 0.16, indicating that system-wide coordination, even when analyzing individual bursts alone, was rare in nerve cords harvested from recovered leeches. When we extended our coordination requirements to include at least three subsequent cycles, the frequency of coordination in the full recovered cords fell to <0.01. Earlier work established that at least three complete crawl cycles with proper periodicity, isochronicity, and intersegmental phase delays were required to deem a neural-activity pattern to be bona fide fictive crawling (Puhl and Mesce 2008, 2010). In our current study, we relaxed the intersegmental phase delay requirements to ensure that we did not eliminate any activity patterns that had established coordination, albeit with greater variability. Our results suggest that even with any type of coordination among the ganglia, in a nerve cord from a recovered leech, the intersegmental coordination of crawl bursting was neither frequent nor sustained through multiple subsequent crawl cycles: essentially it was absent by all standards.

**Figure 4.**
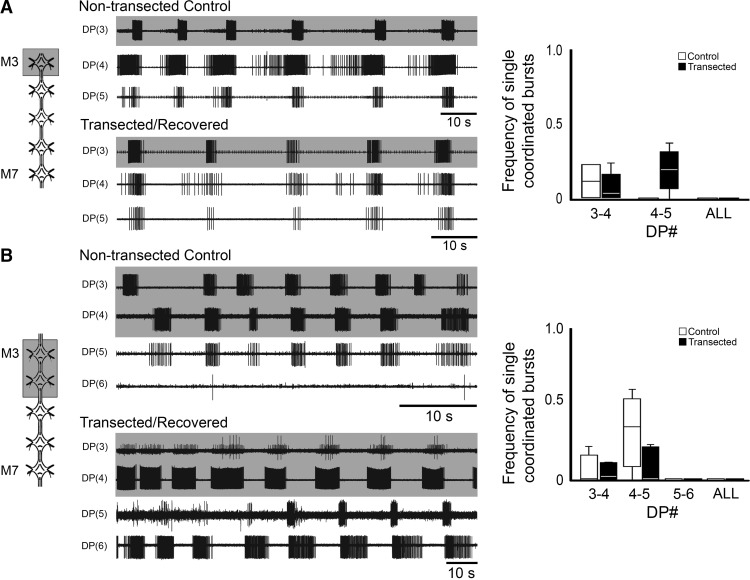
Dopamine-induced rhythmic activity in M3–M7 ganglion chain preparations of transected and recovered leeches. ***A***, ***B***, Left, schematic representations of the preparations showing dopamine-treated ganglia (gray shading). Traces, extracellular recordings from DP nerves in M3, M4, and M5 ganglia. Gray shading denotes dopamine-treated ganglia, which correspond with the schematics. Right, boxplots showing the frequency of single DE-3 bursts exhibiting crawl-specific coordination for pairs of DP nerve recordings of non-transected controls (white boxes) and transected/recovered (black boxes) preparations. Lines within the boxes denote the median, and the box range denotes the 25th–75th percentiles. Error bars denote the entire range of the dataset. ***A***, DP activity from preparations where only M3 was treated with dopamine of a nontransected control (top traces) and a transected/recovered leech (bottom traces). **B**, DP activity of preparations where both M3 and M4 were treated with dopamine of controls (top traces) and transected/recovered (bottom traces) leeches. In DP(6) of the non-transected control, no DE-3 bursts were observed, but occasional DE-3 spikes were generated. All dopamine concentrations were 50 µM.

Based on these results, it seemed that our hypothesis that crawl-specific coordination could arise from plastic changes in the CPGs alone was not supported. Before fully rejecting it, however, we tested an alternative hypothesis based, partially, on a result from Harley et al. (2015), which reported that the “lead” ganglion (closest to the site of transection) likely plays a distinct role in the recovery process because its removal leads to a second round of crawl loss and recovery. Thus, we hypothesized that this lead ganglion may adopt a coordinating role in controlling crawling if it is preferentially activated and permitted to drive its adjacent oscillators, which then can drive each other in the form of a concatenated series of oscillator inputs. To test this idea, we focally applied 50 µM DA to the lead ganglion only. Preparations consisted of 5 connected segmental ganglia, from M3 (the lead ganglion) through M7. These ganglia were obtained from the CNS of control and crawl-recovered leeches. As a baseline for comparison, we focally applied DA to M3 of an M3–M7 preparation, which had not been transected (example, Fig. 4*A*, top traces). Based on similar experiments conducted by Puhl and Mesce (2010), we expected the DA-treated ganglion to exhibit fictive crawling and for the posterior adjacent ganglia to show some sort of crawl-specific rhythmic activity, but lacking intersegmental coordination. In these control preparations, indeed, we observed DE-3 bursting resembling fictive crawling in the DA-treated M3 lead ganglion (top, gray-shaded trace). The period of bursting was 26.30 ± 3.51 s, which is within the normal range for fictive crawling (Puhl and Mesce, 2008). DP recordings of the two adjacent posterior ganglia, M4 and M5, exhibited DE-3 bursting with a crawl-like periodicity as well. The periods for these recordings were 23.53 ± 2.46 s and 18.59 ± 3.55 s, respectively. Among all of our preparations of this type (*n* = 4), crawl-appropriate intersegmental coordination between M3 and M4 was lacking. Crawl-specific single coordinated burst events were observed with a frequency of only 0.11 ± 0.16 (Fig. 4*A*, graph, white boxes). We never observed any crawl-specific coordination that was sustained for more than two consecutive bursts (data not shown). No crawl-appropriate coordination was observed between M4 and M5, therefore never across all the ganglia; thus an essential feature of fully coordinated fictive crawling was absent. Although intrinsic changes in the lead ganglion of recovered animals, theoretically, could have led to intersegmental coordination, the activity patterns we observed in M3–M7 preparations dissected from recovered leeches were similar to those of controls (example, Fig. 4*A*, bottom traces; *n* = 8) and lacked any semblance of sustained crawl-specific coordinated DE-3 activity. The DA-treated lead ganglion exhibited fictive crawling-like DE-3 bursting (top gray-shaded trace), as did the M4 and M5 ganglia. The periods for M3 were 20.23 ± 10.8 s, 24.64 ± 3.6 s for M4, and 23.82 ± 8.46 s for M5. The periods of the controls and the transected/recovered preparations of this type were not significantly different from one another (*p* > 0.5). Again, although a single ganglion (CPG) could induce crawl bursting in adjacent ganglia, crawl-specific coordination was lacking. In M3–M7 preparations, from recovered leeches, the absence of crawl-specific coordination of individual bursts is reflected by a coordination frequency of 0.08 ± 0.10 between M3 and M4, and 0.18 ± 0.15 between M4 and M5 (Fig. 4*A*, graph, black boxes). For even a single crawl cycle, we never observed coordination among all of the recorded ganglia. Although a statistical analysis of DE-3 coordinated events was significantly different between controls and the transected/recovered preparations (between ganglia M4 and M5; *p* = 0.04), this relationship was not maintained when our analysis extended to multiple sequential bursts between these oscillators. Thus this relationship is not particularly noteworthy in the context of whether or not these ganglia (CPGs) are capable of self-organizing their activities into a crawl-specific coordination pattern.

We continued our analysis by treating the anterior-most two ganglia with DA. Our rationale was that DE-3 bursting was exhibited in the anterior three ganglia of the M3–M7 preparations and, perhaps, the system required a larger drive for coordination to self-organize after lost signaling from the brain. We repeated our experiments described above but with focal DA application to both M3 and M4 in 5 ganglia preparations from control and recovered leeches. The top traces of Fig. 4*B* show an example of the neural activity observed in an uninjured control, while the bottom set of traces shows the DP activity from a recovered animal. The DA-treated ganglia (gray shaded traces) of the control animal generated DE-3 bursts with crawl-like characteristics. Collectively, when rhythmic activity was observed, the periods for the control animals were 25.65 ± 4.2 s for M3, 22.17 ± 1.98 s for M4, and 24.87 ± 2.69 s for M5. The periods among these ganglia were not significantly different from one another (*p* > 0.5). Among all the control leeches examined, the frequency of observing single coordinated bursts in these ganglia was 0.07 ± 0.13 (Fig. 4*B*, graph, white boxes). The M5 ganglion, which was the most anterior one that lacked DA exposure, exhibited fictive crawl-like bursting in 4 out of 5 preparations. The frequency of single coordinated bursts between M4 and M5 was 0.32 ± 0.30, which was higher than between the two DA treated ganglia, but not significantly so (*p* = 0.16). The M6 ganglion (in control traces displayed) did not exhibit fictive crawl-like activity, but there was occasional DE-3 activity. Among all control preparations analyzed, there were no coordinated bursts generated between M5 and M6, and we never detected any fictive crawling cycles where all of the ganglia were coordinated in a crawl-specific manner (Fig. 4*B*, graph). Similar to preparations from control leeches, the expression of fictive crawling in both DA-treated ganglia of preparations from recovered animals varied (*n* = 8). In the example shown in Fig. 4*B* (bottom traces), only M4 was generating fictive crawling-like DE-3 bursting. Fictive crawl-like DE-3 bursting was observed in all of the ganglia from which we recorded, but similar to controls, not all of the ganglia produced this activity at the same time. In the example shown, DE-3 bursting was observed in M5 and M6. When DE-3 bursting was observed, the periods were 10.72 ± 1.02 s for M3, 10.78 ± 2.38 s for M4, 12.13 ± 1.14 s for M5. These periods were all within the normal range for fictive crawling and not significantly different from one another or from DA-induced fictive crawling of the control preparations (*p* > 0.5 in all cases). Periodic bursting was observed in M6 in only one preparation and the period was 15.13 s. The frequency of single coordinated bursts between the DA-treated ganglia (M3/M4) was 0.09 ± 0.15 and between M4/M5, the frequency was 0.08 ± 0.12 (Fig. 4*B*, graph, black bars). These results were similar to control preparations, but the occurrences of coordinated events were slightly less frequent. The absence of any coordination between M5 and M6 or among all of the recorded ganglia was also seen in the preparations from recovered leeches.

**Figure 5.**
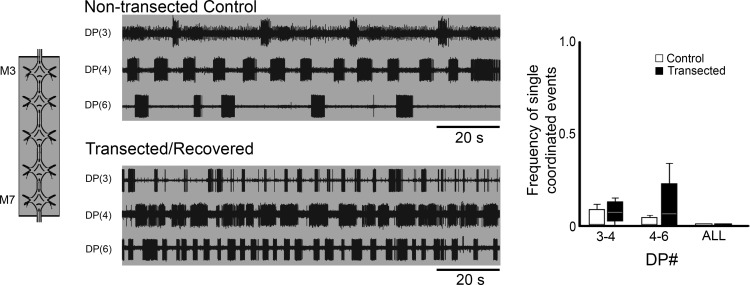
Dopamine-induced rhythmic activity in M3–M7 ganglion chain preparations of transected and recovered leeches. Left, schematic representation of the preparation showing all ganglia were dopamine-treated (50 µm; gray shading). Traces, extracellular recordings of DP nerve of M3, M4, and M6. Top traces show DP activity from a untransected control, and bottom traces are from an M2/M3 transected and recovered leech. Gray shading denotes dopamine-treated ganglia. Right, boxplots showing the frequency of single DE-3 bursts exhibiting crawl-specific coordination for pairs of DP nerve recordings. White boxes are from nontransected controls and black boxes are from transected/recovered preparations. Lines within the boxes denote the median, and the box range denotes the 25th–75th percentiles. Error bars denote the entire range of the dataset.

Our final attempt to elicit coordinated fictive crawling in the 5 ganglia M3–M7 preparations (from recovered leeches) involved applying DA to 3/5 of the ganglia (data not shown; *n* = 2) and on all five ganglia (Fig. 5; *n* = 6). The top set of traces of Fig. 5 were recorded from DP nerves of a control animal (*n* = 3). The resulting uncoordinated DE-3 bursting was similar to that reported previously (Puhl and Mesce, 2010). The periods were 18.94 ± 0.32 s for M3, 28.77 ± 5.48 s for M4, and 27.37 ± 6.60 s for M6. Statistically significant differences in the periods were detected among these recordings (*p* = 0.03); however, we did not perform follow-up analyses as the DE-3 bursts lacked coordination. All of the ganglia were capable of producing rhythmic DE-3 bursting resembling fictive crawling, but the periods were often more variable within a given recording. A similar activity profile was observed in preparations from recovered leeches (Fig. 5, bottom traces). The periods were 14.22 ± 9.68 s for M3, 10.97 ± 0.8 s for M4, and 16.6 ± 7.74 s for M6 (not significantly different; *p* = 0.18). The frequency of single coordinated events between M3 (the lead ganglion) and M4 was 0.03 ± 0.06 for controls and 0.07 ± 0.07 for recovered preparations (Fig 5, graph, white and black boxes, respectively). Between M4 and M6, the frequencies of coordination were 0.02 ± 0.03 for controls and 0.11 ± 0.16 for recovered animals. Similar to the other focally treated DA applications, we never observed any examples of all ganglia displaying crawl-specific intersegmental coordination within the same crawl cycle (either in recordings from control or recovered preparations).

An important finding from our work described above was that individual crawl CPGs from recovered leeches were functional and could be activated by DA as shown previously for uninjured animals (Puhl and Mesce, 2008). Unlike control whole cord preparations, with intact descending cephalic signaling, nerve cords from recovered animals were unable to self-organize to produce anterior-to-posterior directed waves of CPG activation. These results conflict with the fact that intact animals, from which these *in vitro* preparations came, exhibited fully coordinated overt crawling (Fig. 1).

**Figure 6.**
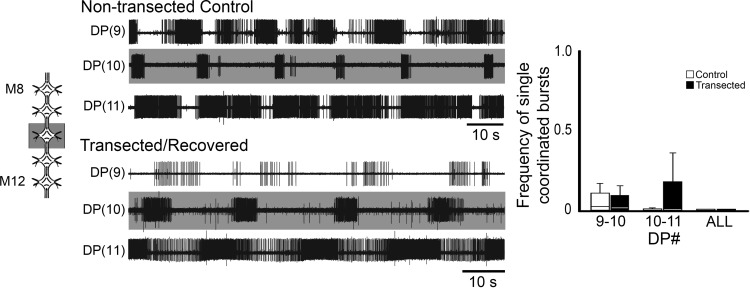
Dopamine-induced rhythmic activity in M8-M12 chain preparations from the middle body region of transected and recovered leeches. Left, schematic representation of the preparations. The middle ganglion (M10) was dopamine-treated (50 µm; gray shading). Traces, extracellular recordings of DP nerves of M9, M10, and M11. Top traces show DP activity from a nontransected control, and bottom traces are from a transected/recovered leech. Gray shading denotes dopamine-treated ganglia. Right, boxplots of the frequency of single DE-3 bursts exhibiting crawl-specific coordination for pairs of DP nerve recordings of nontransected controls (white boxes) and transected/recovered (black boxes) preparations. Lines within the boxes denote the median and the box range denotes the 25th–75th percentiles. Error bars denote the entire range of the dataset.

In the original model for crawl intersegmental coordination (Puhl and Mesce, 2010; Puhl et al., 2012), two essential aspects were needed: the descending signaling from R3b-1 and local excitatory drive from neighboring CPGs. To confirm whether both ascending and descending local oscillator-to-oscillator drive was intact in nerve cords from recovered animals, we repeated experiments done by Puhl and Mesce (2010). In the recovered condition, 5 connected ganglia were dissected and DA was applied to the middle ganglion (Fig. 6). The top set of traces (Fig. 6) show DP activity in a DA-treated ganglion (M10; gray shading) and in the anterior (M9) and posterior (M11) adjacent ganglia of a control leech, while the bottom set shows recordings from a similar preparation of a recovered leech. Fictive crawl-like DE-3 bursting was observed in the DA-treated ganglion of both controls and recovered leeches (Fig. 6, gray shaded traces). In the posterior adjacent ganglion, of both types of preparations, we observed an increase in DE-3 spiking. The frequency of DE-3 spiking was rhythmically modulated with a period consistent with fictive crawling. In the control preparation shown in Fig. 6, DE-3 bursting was observed in the ganglion anterior to the DA-treated one (M9). Among all of the controls, the activity of the anterior adjacent ganglion exhibited rhythmic DE-3 bursting, although the frequency of spiking of DE-3 within a burst varied as did the ratio of the number of bursts compared to the DA-treated ganglia. In these controls, the frequency of observing single bursts with intersegmental coordination appropriate for fictive crawling in the anterior and DA-treated pairs (M9–M10) was 0.05 ± 0.08, and we observed no coordination in the M10–M11 recording pairs (Fig. 6, graph, white boxes). In the anterior adjacent ganglia of recovered leeches, we most often observed rhythmic bursting in DE-3, with lower frequency of spiking compared to normal fictive crawling, but still retaining a periodicity which resembled fictive crawling. With a frequency of 0.05 ± 0.07, we observed coordination of single bursts between M9–M10 and 0.09 ± 0.18 in the M10–M11 pair. The frequency of observing crawl-specific coordinated events was not significantly different when the controls were compared to the transected/recovered recordings (*p* > 0.5). In alignment with Puhl and Mesce (2010) and the results discussed above, we never observed crawl-specific coordination among all of the ganglia recorded in either control or recovered preparations. Furthermore, we were able to confirm that crawl-recovered CPGs retain their ability to drive the crawl CPGs in their adjacent anterior and posterior ganglia as they do in control animals.

The results of our *in vitro* studies described above established that in recovered leeches, the activity of the neural circuitry within the CNS was insufficient to generate sustained intersegmental coordination across multiple ganglia or sustain coordination through multiple cycles, as is the case in uninjured leeches where descending information from R3b-1 is present. Thus the most logical factor to consider next was sensory information, which might supply the timing cues essential for intersegmental coordination.

**Figure 7.**
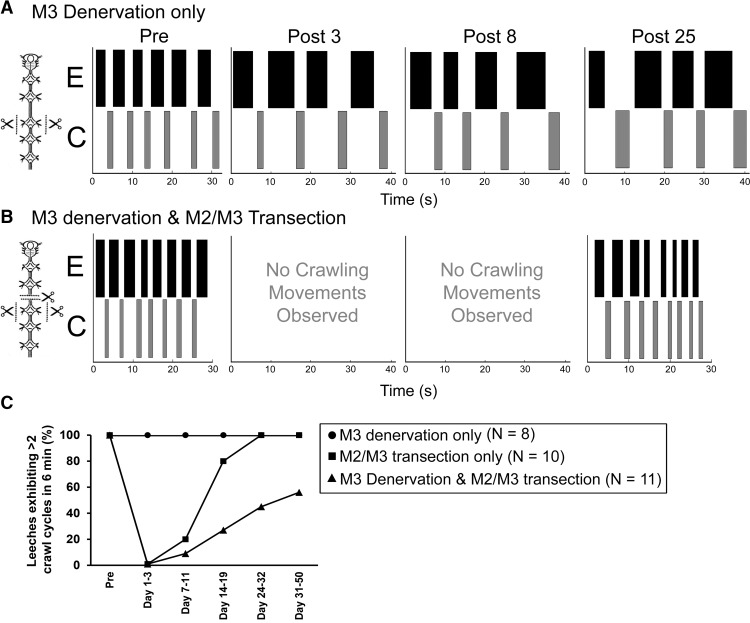
Overt crawling behavior after a surgery combining denervation of the lead ganglion (M3) with a complete transection of the M2/M3 interganglionic connectives. ***A***, ***B***, Movement rasters depicting elongation (E, black boxes) and contraction (C, gray boxes) movements over time for an individual receiving an M3-denervation only surgical procedure (***A***) and a leech undergoing M3-denervation combined with M2/M3 connective transection (***B***). Movements are shown for time points before the surgery (Pre), and 4, 8, and 25 days after surgery. ***C***, Graph showing the percentage of leeches exhibiting crawling behavior for M3-denervation only (*n* = 8), M2/M3 transection only (*n* = 10), and M3 denervation combined with M2/M3 transection (*n* = 11). Additional quantitative analyses are presented in the body of the Results section.

In our next series of studies, we argued that if afferent input were important for crawl recovery then its removal would perturb the leech’s ability to display overt coordinated crawling. We also integrated this idea with the known role of the lead ganglion in the recovery process (Harley et al., 2015). Thus we generated a series of nerve cord transections as before (M2/M3) while also completely severing all the nerve roots associated with the lead ganglion M3 (both anterior and posterior roots, bilaterally). To determine if severing the nerve roots impacts a leech’s ability to crawl, we first needed to perform similar nerve-root transections on M3 in control leeches (schematic, Fig 7*A*, left, *n* = 8). We observed that this procedure did not impede the leech’s ability to generate productive and coordinated crawling movements. We compared the elongation, contraction, and cycle durations of control and denervated leeches to determine if the behaviors were different. For controls, the mean elongation, contraction, and cycle durations were 4.4 ± 0.8 s, 1.4 ± 0.1 s, and 6.6 ± 1.2 s, respectively (*n* = 8). For denervated leeches (38 days post-surgery) the elongation duration was 2.9 ± 0.1 s, the contraction duration was 1.3 ± 0.1 s, and the cycle period was 4.9 ± 0.5 s (*N* = 8). No significant differences existed between the control and denervated animals for any of the movements analyzed (*p* > 0.5 for elongation and contraction movements and *p* = 0.22 for the cycle periods). We performed an additional analysis similar to Harley et al. (2015) to determine the proportion of leeches exhibiting an anterior-to-posterior propagation of crawl movements by determining the activation sequence of body quadrants. All of the control (*n* = 11) and denervated (*n* = 8) leeches exhibited sequential caudally directed activation of the body quadrants for all crawl cycles (data not shown). An example of the movements of one leech is shown in Fig. 7*A* (rasters). Normal crawling movements were observed before surgery (Pre), and 4, 8, and 25 days after M3 nerve-root denervations. In fact, all of the eight leeches examined produced at least three cycles of overt crawling during all time points (Fig. 7*C*). Thus, when central connections remained intact, removal of a segment’s worth of sensory or motor signaling did not impact the dynamics of crawling.

To determine if depriving the lead ganglion of sensory information affected crawl recovery, we performed surgeries to sever the nerve roots of the lead ganglion (i.e., M3) and transected the M2/M3 connectives (*n* = 11). Fig. 7*B* shows the elongation and contraction movements of a leech receiving this surgical manipulation. Fig. 7*C* reports the percentage of leeches that produced three or more cycles of crawling for several time points. We analyzed the following sets of leeches: those that received no M2/M3 transection, but had the M3 roots denervated; ones that received a M2/M3 transection with no nerve root denervation; and ones receiving both M3 root denervation and the M2/M3 transection. Before surgery, all leeches readily crawled. Immediately after surgery and for several days afterward, all leeches that were transected did not express any crawling. By days 7–11, at least one individual from both the transected-only and denervated/transected leech groups began crawling. By days 24–32, the transected-only leeches all were crawling (*n* = 10), while only ∼40% of the denervated and transected leeches were crawling. At day 50, only ∼55% of the dually denervated and transected leeches had recovered, indicating that denervating the nerve roots of the lead ganglion negatively affected crawl recovery. Of these leeches that eventually exhibited recovered crawling, the elongation and contraction durations were 4.9 ± 1.3 s and 2.8 ± 0.7 s, respectively. The cycle periods were 10.2 ± 2.3 s. No significant differences existed between the duration of crawl movements in the dually transected and denervated groups and the nontransected controls (*p* > 0.5 for elongation duration, *p* = 0.15 for contraction duration, and *p* = 0.22 for cycle period). Lastly, we measured the ability of leeches to display activation of body quadrants in a caudally directed sequence. Dually transected and denervated leeches produced properly sequenced body movements 93.3% ± 4.1% of the time (*n* = 11 leeches; 8–15 cycles per leech analyzed).Thus their recovered crawling appeared normal the vast majority of the time.

**Figure 8.**
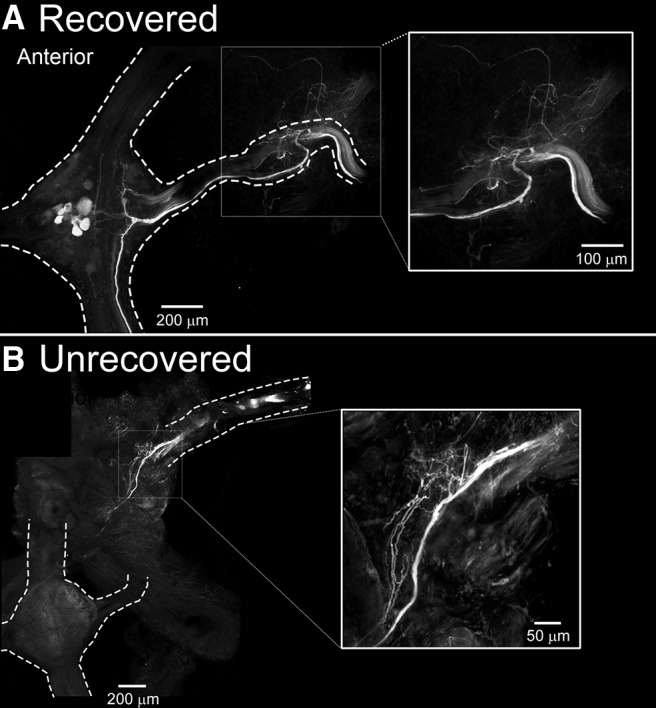
Laser-scanning confocal micrographs of Cy3-labeled Neurobiotin nerve-root back-fills of the lead ganglion (M3) from transected and denervated leeches that recovered (***A***) and did not recover (***B***). ***A***, Micrograph of the lead ganglion (M3) of a leech that received a surgery combining M3-denervation and M2/M3 connective transection and exhibited recovered crawling behavior. Back-fill procedure was performed 29 days posttransection (8 days postrecovery). Right, inset is a magnified view of the gray box. ***B***, Micrograph of the lead ganglion (M3) of a leech that received the same M3 nerve-root denervation and M2/M3 connective transection surgery, but did not recover crawling after 40 days. Right, inset is a magnified view of the gray box. Dotted lines in ***A*** and ***B*** denote edges of the M3 ganglion, connectives, and nerve roots.

To verify whether our nerve root denervations prevented nerve regrowth into the lead ganglion, we backfilled the peripheral nerves with Neurobiotin at sites far distal to the original location of surgery. This procedure yielded some surprising results. When peripheral M3 nerves in the body segments were back-filled, in animals exhibiting delayed crawl recovery (M3 denervation and M2/M3 transection), we observed the labeling of sensory nerve terminals and motoneuronal somata with Neurobiotin within M3 (Fig. 8*A*; *n* = 4 of 7 backfilled preparations). Initially, we assumed that our nerve-root cuts were incomplete, but of the 4 preparations backfilled, 3 showed evidence that labeled peripheral fibers were able to grow past the scar tissue, entering their formerly targeted (home) ganglion. Importantly, no evidence of M3 cell labeling occurred in leeches that did not exhibit crawling after 30–50 days (*n* = 3 of 7 backfilled preparations). In all backfills of both recovered and unrecovered leeches, extensive outgrowth of Neurobiotin-labeled fibers was observed near the site of denervation (Fig. 8*A*, *B*, insets), but only growth that made it into the CNS was correlated with crawl recovery.

## Discussion

### Overview

In this study of the medicinal leech, we looked at the recovery of crawling after removing descending information from the brain, which was shown previously to be vital for crawl-specific intersegmental coordination (Puhl and Mesce, 2010; Puhl et al., 2012). Our approach was fairly exhaustive to establish whether individual crawl central pattern generators (CPGs) were able to coordinate with each *in vitro* to generate a caudally directed metachronal wave of fictive crawl activity, effectively matching the animal’s overt coordinated output. Although we determined that the crawl CPGs retained their sensitivity to DA after injury and individual ganglia were competent to exhibit fictive crawling, none of the CPGs were able to direct intersegmental coordination on their own (Fig. 3) despite retaining local oscillator-to-oscillator drive in both the anterior (Fig. 6) and posterior (Figs. 4 and 6) directions. Because overt recovered crawling was essentially indistinguishable from control animals (this study and Harley et al., 2015), we can conclude that sensory information from the body is key to the leech’s recovery of crawl-specific intersegmental coordination. Our final experiment to disrupt the flow of afferent input to the anterior-most ganglion (directly below the nerve-cord transection site) indicated that afferent input was indispensable for the crawl-recovery process (Fig. 7).

Previous studies have shown that, under idealized conditions, CNS connections can regrow and reconnect (for example, Nicholls, 1987), but in adult leeches, severed CNS fibers do not exhibit axonal outgrowth or reconnect *in situ* (Harik et al., 1999). In contrast, peripheral fibers can regrow across an injury site and in some cases reestablish functional connections within a segmental ganglion (van Essen and Jansen, 1977). Here, we have shown a correlation between crawl recovery and the regrowth of peripheral fibers (Fig. 8). Our preliminary data suggest that the body-wall proprioceptive stretch receptors, specifically, are vital to crawl recovery (Fig. 8 and Puhl, Bigelow, Mesce, personal observations).

### Intersegmental coordination for locomotion in the leech

Locomotion in the leech involves two forms: an aquatic one comprising swimming and a terrestrial form studied here, crawling. The circuitry for swimming and crawling are shared at many levels, including interneurons, motoneurons, and sensory neurons (Esch et al., 2002; Briggman and Kristan, 2006). For both modes of locomotion, the propagation of a coordinated metachronal wave is directed caudally along the nerve cord and body. There are, however, a number of important functional-design features that differentiate the two locomotor forms: mainly, swimming is a fast rhythm and does not require descending information for its intersegmental coordination (Brodfuehrer and Friesen, 1986; Puhl et al., 2012). Additionally, because swimming becomes disinhibited swim maintenance is overly expressed after a nerve cord transection in behaving animals (Harley et al., 2015). Thus leeches do not need to recover their ability to swim, although its spontaneous nature is eventually modulated during the crawl-recovery process (Harley et al., 2015). Essentially, the swim CPGs are capable of producing fictive swim-related intersegmental phase relationships in the absence of sensory input (Kristan and Calabrese, 1976) and descending information from the brain (Brodfuehrer and Friesen, 1986; Puhl and Mesce, 2010). Another important functional difference involves the excitability of the CPGs themselves. Each of the 21 midbody ganglia contains a complete CPG for crawling and each one can equally be activated independently via application of DA (Puhl and Mesce, 2008). Although present in all segmental ganglia, the swim CPGs are functionally distinct (Hocker et al., 2000). In isolation, CPGs housed in the middle-body region can be activated most easily with either serotonin or an electric shock. It is much more difficult, however, to activate the swim CPGs in the anterior ganglia and not at all possible to activate the posterior ganglia *in vitro*. Sensory inputs to the CPGs arising from stretch receptors provide the additional excitation and coordination needed to maintain swim activity through these posterior ganglia (Cang and Friesen, 2002).

A further point regarding swimming is that the number of connected swim CPGs (i.e., ganglia) can influence the intersegmental phase delays—a larger number of attached ganglia participating in the swim network leads to intersegmental phase delays that more closely resemble those observed during intact swimming (Pearce and Friesen, 1985). In our experiments, we activated an increasing number of crawl CPGs in nerve cord chains, using DA, but did not see any changes in crawl-specific intersegmental coordination when one (Fig. 4*A*), two (Fig. 4*B*), five (Fig. 5), or all (Fig. 2) of the ganglia in a chain (from a recovered leech) were induced to crawl. Essentially, we saw no evidence of oscillator-to-oscillator coupling that could produce a coordinated metachronal wave of crawl locomotor activity, in sharp contrast to the capabilities of the central coupling of the swim CPGs and their ability to generate a metachronal wave along chains of isolated ganglia completely devoid of descending and afferent information.

These differing functional architectures, across the two forms of locomotion, provide a rich platform for understanding the biological constraints and perhaps advantages for why, after injury, crawl-related circuits become completely reliant on sensory input for their intersegmental coordination. Ironically, the swim circuits, but not the crawl ones, seem to depend on sensory feedback (stretch receptors) to slow their centrally generated phase lags to produce their one-wavelength body form during swimming (Cang and Friesen, 2000, 2002). During normal overt crawling, which is a much slower behavior compared to swimming (Stern-Tomlinson et al., 1986), there is no evidence that sensory feedback is required for intersegmental coordination, although further studies need to be conducted. Our results further support this idea, as we observed no significant changes in the coordination of overt crawling when ganglia were deprived of sensory input (Fig. 7*A*). Our biologically based working models of intersegmental coordination also do not incorporate the need for sensory feedback to generate intersegmental coordination (Puhl and Mesce, 2010; Puhl et al., 2012). The problem of how a centrally generated coordination system can adopt the use of afferent information to set the phase coupling of its oscillators after injury is, indeed, a fascinating topic for future study.

### Pulling it together—central and peripheral contributions to locomotion across animal systems

Our results suggest that crawling, in the recovered state, is likely generated via CPGs and a combination of both local excitatory drive and afferent information. This configuration is a substantial change from the uninjured state, where coordination arises through contributions from local excitatory drive and descending command signals (Puhl and Mesce, 2010; Puhl et al., 2012). An important and open question to address is which types of afferent information are involved in crawling or its recovery. During swimming, the ventral stretch receptors are rhythmically active and influence various swim-related parameters such as phase coupling (Cang and Friesen, 2000). In fact, when nerve cord transections are made in connectives between two segmental ganglia, deficits in CPG function are immediately compensated for by stretch receptors within body segments adjacent to the site of central injury (Yu and Friesen, 2004). Stretch receptors involved in swim coordination are very likely the same ones that become involved in crawl coordination during the recovery process. In the Neurobiotin-labeled sensory fibers that we observed, terminals of in-growing stretch receptors similar to those described by Fan and Friesen (2006) were visible ipsilateral to the site of tracer placement (Fig. 8*A*). Because the mechanosensory pressure (P) and touch (T) sensitive neurons have their somata located centrally, the body-wall stretch receptors are most likely the source of afferent information needed for crawl recovery.

By definition, a CPG generates patterned motor activity without the need for sensory inputs or feedback (Marder and Calabrese, 1996). Some animal systems, however, heavily rely on afferent information for the functional coordination of locomotor CPGs even when uninjured. For example, coordination of the intra-leg joints during walking in the stick insect critically relies on both central CPG activity and sensory input from the legs (Büschges, 2005; Mantziaris et al., 2017). Each joint of the middle leg is controlled by a CPG which can function independently (Bässler and Büschges, 1998). The load-sensing proprioceptive sensory neurons can access the state of the CPGs and are involved in transitions throughout the step-cycle (Akay et al., 2004). In the nematode, *Caenorhabditis elegans*, proprioceptive inputs into the CPGs are necessary for coordinating and propagating rhythmic muscle contractions during forward locomotion (Wen et al., 2012). In mammals, load signals from proprioceptors are sufficient to induce the transition from one phase of the step-cycle for locomotion to the next (Grillner and Rossignol, 1978; Hiebert and Pearson, 1999). Modeling studies of salamander (Harischandra et al., 2011) and mammalian (Rybak et al., 2006) stepping have suggested that proprioceptive inputs, although not absolutely essential for the behavior, likely play a key role in fully functional locomotion. Collectively, these studies provide further justification to hypothesize that the source of sensory information, which seems to compensate for the lack of descending inputs, arises from proprioceptors—the potential roles of such proprioceptors for crawling in the uninjured state is currently under investigation.

### Locomotor recovery across taxa

Our results share a number of aspects with spinal cord injury (SCI) and its recovery in mammals. After SCI, locomotor behaviors are disrupted; however, the locomotor CPGs appear to remain functional after partial or complete transection of the spinal cord. In acutely paralyzed and spinalized cats, whose sensory spinal roots are severed, fictive locomotor-like activity is observed in nerve recordings after treatment with pharmacological agents, although normal weight-bearing stepping is not observed in these animals (Grillner and Zangger, 1979). Spontaneous fictive locomotion is seen in chronically spinalized cats (i.e., cats with SCI) incapable of independent walking as well (Pearson and Rossignol, 1991).

Similar to our results, some locomotor function can be restored after SCI, in mammals, when afferent inputs are present and active (Rossignol et al., 1996; Figs. 1 and 7). In chicks, after incomplete SCI, phasic sensory feedback to spinal circuits facilitated the recovery of locomotion (Muir and Steeves, 1995). After weeks to months of treadmill training, adult cats can exhibit limited locomotor abilities including weight-bearing hind-limb stepping (Barbeau and Rossignol, 1987). When peripheral afferent fibers from the ankle of the hindlimb are denervated and the spinal cord is transected, subsequent locomotor recovery is blocked even with treadmill training (Carrier et al., 1997). These results are strikingly similar to the blockade of locomotor recovery we observed when nerve roots were severed (Fig. 7*B*). Denervation of ankle afferent fibers alone, again in adult cats, had only a subtle effect on locomotion and did not prevent productive stepping. These results also complement our studies where we cut the nerve roots in an uninjured control leech (Fig. 7*A*). Furthermore, in rats, treadmill and swim training, after an incomplete SCI, have led to the partial recovery of weight-bearing stepping (Smith et al., 2006). In rats with a complete transection of the spinal cord, greater degrees of locomotor recovery were seen when treadmill training was supplemented with robotic devices, which increased proprioceptive feedback to the spinal CPGs as compared to treadmill training or no training (Cha et al., 2007). Lastly, in humans with SCI, phasic afferent stimulation via robotic devices (Wirz et al., 2005; Cheung et al., 2017) or electrical and pharmacological stimulation (Minassian et al., 2017) has facilitated the recovery of stepping ability.

In the lamprey, a transection of the cord causes a paralysis below the site of lesion (McClellan, 1994). Within about 2 weeks, however, locomotor behavior begins to recover nearest to the site of lesion followed by activity within more distal segments. Qualitatively, these results show a similar time course of recovery to our own work when the segment housing the lead ganglion is denervated and crawling becomes delayed or blocked (Figs. 1*B* and 7*B*). Decades of work using lampreys (Parker, 2017) has established that recovery of locomotion after complete spinal transection is mediated primarily by regeneration of the central connections across the site of injury (McClellan, 1994; Kiemel and Cohen, 1998). Although comparisons of central regrowth is not applicable to our studies (Harley et al., 2015), the timing of the regrowth of sensory neurons clearly is.

Even when regeneration is possible, as in the lamprey, neuronal connections must be reintegrated within spinal locomotor networks correctly (Parker, 2017), and plastic changes within locomotor networks need to accommodate proprioceptive inputs to mediate successful recovery. In recovered leeches, the anterior-most ganglion closest to the site of injury (“lead” ganglion) appears to be a critical factor in the recovery process because its loss results in the need for a second round of recovery (Harley et al., 2015); importantly, its denervation prevents recovery as shown here (Figs. 7 and 8).

Finally, likely all locomotor recovery processes require some form of homeostatic plasticity in the synaptic connections, neuronal membrane properties and neuromodulation of recovering networks (Sharples et al., 2014). In the mollusc, *Tritonia diomedea,* for example, disruption of the CNS by chemical blockade or transection of connectives results in the loss of escape swimming followed by swim recovery (Sakurai and Katz, 2009). In this example, recovery is achieved through plastic mechanisms involving novel polysynaptic pathways becoming established within the CNS (Sakurai et al., 2016). The crustacean stomatogastric ganglion also provides an excellent example of how the elimination of higher-order modulatory inputs causes a disruption in CPG function, with recovery based on changes in new ion-channel expression patterns and function (Mizrahi et al., 2001; Thoby-Brisson and Simmers, 2002; Khorkova and Golowasch, 2007; Temporal et al., 2014) or alterations in metabotropic receptor function (Lett et al., 2017).

How the central and peripheral nervous systems become retuned and work together to establish functional crawl patterns will clearly be facilitated by the experimental tractability of the leech nervous system. The ability to study identifiable neurons, with known contributions to specific motor actions (Kristan et al., 2005), will be extremely useful in understanding the process of functional recovery after CNS injury and, hopefully, will provide a guide for future studies of locomotor recovery in vertebrate animals. The work we have presented here clarifies that CPG neural networks are highly plastic in their ability to incorporate different timing elements (i.e., proprioceptors) for their intersegmental coordination—how these new elements “know” how to substitute for their former coordinating inputs (i.e., interneuron, R3b-1) remains a fascinating problem for future study.

## Synthesis

Reviewing Editor: Elizabeth Cropper, Mt Sinai Sch Med Decisions are customarily a result of the Reviewing Editor and the peer reviewers coming together and discussing their recommendations until a consensus is reached. When revisions are invited, a fact-based synthesis statement explaining their decision and outlining what is needed to prepare a revision will be listed below.

The reviewers found this to be a study of broad interest since there are obvious parallels to work in other systems. The only major concern related to the statistical analyses (as is described in more detail below). Other comments were largely minor.

Reviewer #1

This is a very interesting paper. By documenting the ability of the peripheral nervous system to mediate motor recovery following injury the authors have opened a new line of research in functional recovery of motor behaviors.

Comment on Statistics

My biggest criticism is the under-utilization of statistical analyses throughout the manuscript. This is especially a concern in Fig. 7 where it would be good to have more than a qualitative judgement that crawling is unchanged in the denervated condition (Fig. 7A) or has completely recovered following denervaton+transection. As it stands the reader only has the representative data in Figs 7A & B to judge level of recovery. This is despite the fact that the authors say in lines 553-554 that they “observed no significant changes in the coordination of overt crawling when ganglia were deprived of sensory input (Fig. 7A)”. Surely there are quantifiable elements of the behavior that could be statistically analyzed to determine whether crawling prior to lesioning is the same after lesioning or after lesioning and recovery. Possible measures to put into a statistical analysis would seem to include the number or frequency of crawl cycles, duration of elongation and/or contraction phases, duration of crawl, etc.. Other stat related issues that the authors should address are as follows.

a. The authors refer to the use of non-parametric statistical tests, but do not actually state which test they use.

b. Figure 3B data: There seems to be a statistical test between the lesion-adjacent ganglion and the ganglion farther away (period duration data, line 278), but no comparable statistical analysis for the control vs. the recovered groups.

c. Lines 347- 349: No statistical analysis to support that the burst frequency between M4 and M5 was not “significantly different” (Fig. 4B). In fact there seems to be no statistical analysis of the Fig. 4 data.

d. Are the lines in the Fig. 1C scatter/line plot standard deviation or standard error? Please include standard deviation/error lines in Fig. 7C as was done for Fig. 1C. Is there any reason why the data in Figure 1C and 7C cannot undergo statistical analysis? I realize that the 1C data is pretty obvious, but it would be good to confirm statistically that there is a difference in the rate of recovery in Fig. 7C.

Other comments

Could the authors consider and address an alternative explanation for why the isolated cords with no R3b-1 input do not produce coordinated crawling? Perhaps there are elements in the CNS that can reorganize after injury and take on CPG coordination functions, but are simply insensitive to DA. Perhaps they lost sensitivity to DA as a consequence of the injury or they were never sensitive to DA in the first place. This explanation would seem to be a possibility given that no other stimulus besides DA application was used to elicit crawling.

Minor Comments

Line 8: Define “DA”

Line 211: Should this be “SEG and M1”?

Figure 3 results - The authors should report the duration until recovery (range of days) as was done for the Fig. 1 data.

Line 11 & Line 531: In line 11 the authors refer to the CPG as though it were an anatomical unit. In line 531, they say this again but make it clear that in this context CPG=ganglion. They should do this in line 11 as well.

Reviewer #2

This is a very thorough study examining the role of sensory afferents in the recovery of crawling movements in the leech. After transecting the ventral nerve cord, leeches can recover this behavior after a prolonged period. Recordings of 'fictive' crawling evoked by local and global dopamine applications effectively rule out compensatory changes in circuits within the nerve cord, since the output does not resemble the coordinated crawling movements observed in the behaving animal. Instead, lesioning of sensory afferents has the most dramatic effect on crawling among animals that recover this behavior. The authors also demonstrate that recovery is correlated with regeneration of afferents that reach the ventral nerve cord. Overall, the work provides convincing evidence that the recovery of crawling after transection is reliant on sensory afferents which enable central circuits to operate properly. There are obvious parallels to lesions in other model systems, so this provides a new model to investigate locomotor network plasticity in the face of damage. I have only a couple minor queries.

1) For the uninitiated, it would be nice to have an introductory figure outlining the anatomy covered in the manuscript. There are cartoons of the ganglia in later figures that are really helpful, but some of the references to anatomical regions (e.g., L211 SEG), were unclear to me. This also becomes problematic when referring to specific neurons versus nerves versus ganglia (e.g., there are references to motor neuron DE-3 and also DP but the cartoons are labeled M8).

2) Given that the emphasis is on coordination, it's not clear to me why phase relationships between the ganglia are not considered. Instead the focus for analysis is on frequency or cycle period. Some justification for this or additional analysis of phase seems warranted. Apologies if I've missed something here.

3) Were statistics run on the data presented in the figures? If so, it would be good to include some indication of significance there.
